# Localization of Insertion Sequences in Plasmids for L-Cysteine Production in *E. coli*

**DOI:** 10.3390/genes14071317

**Published:** 2023-06-22

**Authors:** Kevin Heieck, Thomas Brück

**Affiliations:** School of Natural Sciences, Technical University of Munich, Lichtenbergstraße 4, 85748 Garching, Germany; kevin.heieck@tum.de

**Keywords:** l-cysteine, *E. coli*, insertion sequence elements, plasmid deep sequencing, metabolic engineering, target site duplications

## Abstract

Insertion sequence elements (ISE) are often found to be responsible for the collapse of production in synthetically engineered *Escherichia coli*. By the transposition of ISE into the open reading frame of the synthetic pathway, *E. coli* cells gain selection advantage over cells expressing the metabolic burdensome production genes. Here, we present the exact entry sites of insertion sequence (IS) families *3* and *5* within plasmids for l-cysteine production in evolved *E. coli* populations. Furthermore, we identified an uncommon occurrence of an 8-bp direct repeat of IS5 which is atypical for this particular family, potentially indicating a new IS5 target site.

## 1. Introduction

Mobile genetic elements, also known as transposable elements, are segments of genetic material capable of relocating within a chromosome and being transferred between chromosomes, plasmids, bacteria and even across different species. Thereby, insertion sequence elements (ISE) are the most ubiquitous mobile genetic elements in bacterial genomes and play a central role in mediating large variations in bacterial genomes. Indeed, among the functional classes found in both prokaryotic and eukaryotic genomic and metagenomics public databases, proteins annotated as transposases or with related functions stand out as the most abundant [1]. With a compact size of 0.7–2.5 kb in length and a *cis*-acting site upon which the transposase acts, they are the simplest type of bacterial transposable element and usually encode for a gene required for transposition [2]. Transposition can occur in copy-paste or cut-paste mechanisms, whereas IS loss is very rare and seems replicative [3]. Cis-sites usually consist of inverted terminal repeats of a few dozen base pairs. In addition, most ISE create short target site duplications (TSD) during the insertion process.

Although it is detrimental in nature to overcome changing environmental conditions by means of evolutionary adaptation, this ability poses a hindrance in the large-scale production of chemicals utilizing microorganisms. The often metabolically burdensome production drives populations to escape mechanisms. Within 60–70 generations, mutations can accumulate in the genome, giving cells a fitness advantage at the expense of production [4,5]. Thereby, mutations did not involve single-nucleotide polymorphisms but rather insertions of ISE into critical regions of synthetic plasmid constructs [4,5,6,7]. Insertions within genes can lead to mutations that result in loss-of-function. On the other hand, insertions between genes have the potential to disrupt the function of promoters or even induce the up-regulation of neighbouring genes in instances where the IS element contains a promoter that faces outward. The frequency of transposition is influenced by various parameters, including growth phase, medium composition, oxygen levels and the structural characteristics of target sites [8]. Among these, however, metabolic stress appears to be a significant driving force behind transposition events.

We recently discovered an accumulation of predominantly IS*3* and *5* reads in plasmids designed for l-cysteine production in evolved *E. coli* populations [5]. These insertions were most likely driven or accelerated by the disruption of sulphur and/or l-cysteine homeostasis within *E. coli* cells. Phenotypically, evolutionary adaptation was observed through increasing growth rates and simultaneously decreasing l-cysteine yields within 60 cell generations. These phenotypic observations were accompanied by genetic mutations, specifically an accumulating number of insertion sequences. Here, we demonstrate that IS*3* and IS*5* transposition were stochastically distributed in open reading frames and in the backbone of plasmids. Moreover, we detected an accumulation of an 8 bp IS*5* target site duplication which is unusual for this particular family.

## 2. Materials and Methods

### 2.1. Plasmid Deep Sequencing

The three plasmids (pCYS, pCYS_i and pCYS_m), designed to enhance l-cysteine yield and propagated for 60 cell divisions in *E. coli* W3110, were isolated using a standard plasmid extraction kit. Eurofins Genomics (Ebersberg, Germany, GmbH) performed subsequent library preparation and deep sequencing using the Illumina NovaSeq 6000 S4 paired-end 2 × 150 bp platform, achieving a per base coverage depth of over 140,000× (Supplementary Figure S1). To ensure high-quality bases, adapter trimming, quality filtering and per-read quality pruning were performed. The reads were then aligned to the corresponding reference plasmid sequence with the Burrows–Wheeler Aligner (BWA). Any reads that could not be mapped to the plasmid reference sequences were aligned to an insertion sequence database (ISfinder_Nucl) using the BWA [9]. The reads that could be mapped to insertion sequences were then compared to each insertion sequence family in the *E. coli* W3110 genome using NCBI’s megablast algorithm (Supplementary Figure S2). Only highly similar sequences (alignment scores > 99.5%) were retained and presented.

### 2.2. Localization of IS Target Site Duplications within Plasmids

The Artificial Transposon Insertion Site Tracker software (Genome Artist, version 2.0) was utilized to pinpoint target site duplications, which are indicative of IS insertion, by identifying reads that contain duplicated plasmid sequences [10]. Reads that could be at least partially mapped to insertion sequence elements 3 and 5 were selected as queries, and alignments were conducted against plasmid sequences pCYS, pCYS_i and pCYS_m. Interval extension was set to short and the advanced extension parameters to default with zero offset = −5, match score = 4, mismatch score = −2 and length modifier = 2. Only target site duplications with the highest mapping scores were selected, and the exact insertion sites were determined for the genes directly impacted by the transposon insertion, down to the nucleotide level.

## 3. Results and Discussion

We recently uncovered that metabolic burden, particularly the interference with sulphur and l-cysteine homeostasis throughout the production of l-cysteine using plasmid systems, exerts a direct negative influence on growth rates and subsequently affects l-cysteine yields. These phenotypic effects were observed in combination with genetic errors which accumulated over short periods (60 generations) through genetic adaptation in the form of insertion sequence (IS) elements. Therefore, a precise localization of insertion sequence elements within critical l-cysteine pathway genes in plasmids should be conducted. Following the identification of predominantly IS*3* and IS*5* reads in plasmids derived from evolved *E. coli* W3110 populations, our aim was to accurately determine the insertion sites. This should elucidate the impact of metabolic stress during l-cysteine production on plasmid stability. In this study, an approach based on the emergence of target site duplications (TSDs) or direct repeats (DRs) after IS integration was employed.

**Figure 1:**
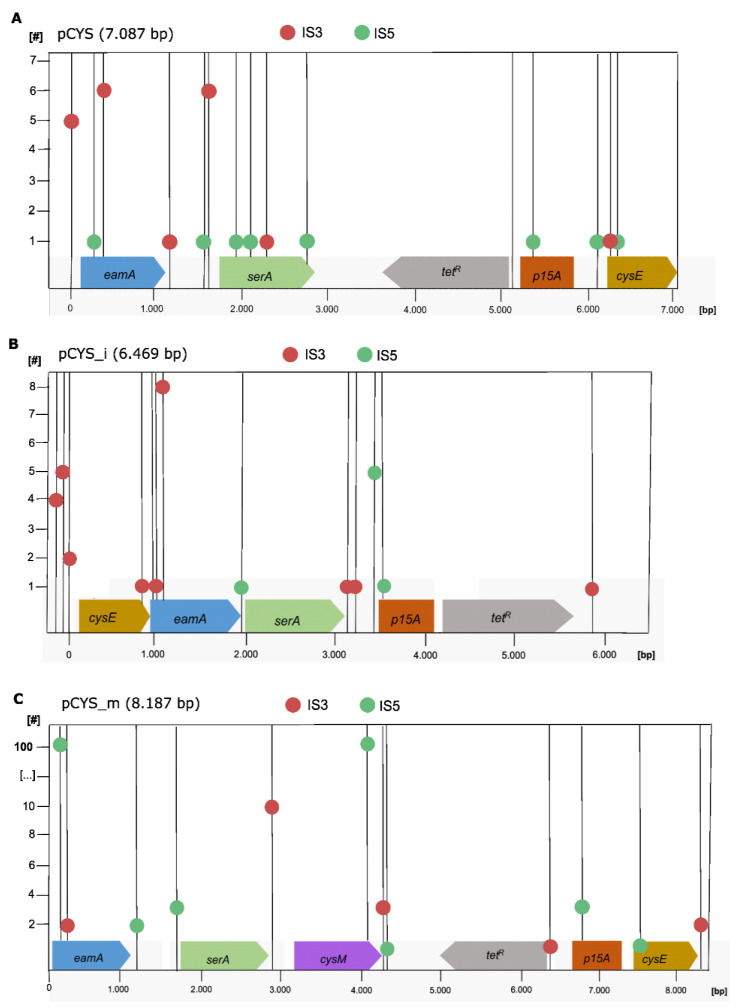
Localization of IS entry sites in plasmids from late W3110 populations. The entry sites of families 3 (indicated by red dots) and 5 (indicated by green dots) were identified and quantified through target site duplications resulting from transposition events in pCYS (**A**), pCYS_i (**B**) and pCYS_m (**C**). The analysis was performed using the Genome ARTIST (Artificial Transposon Insertion Site Tracker), version 2.0 software [10].

The Genome ARTIST software (version 2.0) was employed to identify TSDs. This program allows for the precise determination of the transposon insertion site, the affected gene and the neighbouring genes in close proximity to the insertion sequence (Figure 1). Additionally, the number of specific target site duplications at a particular sequence locus was quantified.

Overall, transpositions of IS*3* and IS*5* were observed to be dispersed throughout the entirety of the plasmid sequences. Target site duplications were identified in various locations, including the plasmid backbone, l-cysteine pathway genes, promoter regions, as well as within the propagation region p15A. It appears that transposition events of these insertion sequence families occurred in a rather stochastic manner, without a strong dependence on specific target sequences.

The prevalence of IS*3* and IS*5* sequences derived from plasmids in the evolved *E. coli* W3110 population aligns with their high representation within the genome of *E. coli* K-12 W3110, with six and ten copies, respectively. Consequently, IS3 emerges as a notably prevalent and extensively distributed IS family [11]. This notable abundance of IS3 in plasmid sequences finds support in the heightened expression of *insJK*, a gene encoding a transposase belonging to the IS*3* family, observed in later-generation populations of W3110 [5].

Upon integration, IS*3* elements create duplications of the target site, typically spanning 3–5 base pairs. Specifically, IS3 features two adjacent reading frames, namely OrfA and OrfB, which partially overlap and are shifted in reading frames −1 and 0, respectively [12]. The synthesis of both the upstream element OrfA and the actual transposase OrfAB occurs, with the latter being a fusion protein that undergoes activation through a programmed translational frameshifting mechanism. OrfA harbours a unique helix-turn-helix (HTH) motif believed to facilitate specific binding to the terminal inverted repeats of OrfAB transposases [13]. Downstream of the HTH motif, at the C-terminus, a conserved leucine zipper (LZ) motif is present, associated with the multimerization of the protein [14]. The OrfAB fusion protein possesses an additional DDE motif, similar to retroviral integrases, which catalyses the transposase reaction [15]. The transposition process follows a “copy-out-paste-in” mechanism, where the original site is retained, and a double-stranded circular DNA intermediate is exploited. This organization is observed in numerous other members of the IS*3* family [16,17]. The rate of frameshifting can vary among different elements, with IS150 approximately exhibiting a 50% frameshift rate [18].

However, with the exception of IS911 and IS150, no specific insertion preferences were observed within the IS*3* family. This is evidenced by the unsuccessful endeavour to identify specific sequence patterns at IS*3* insertion sites in the conducted study. Consequently, the unpredictable nature of these insertions makes it impractical to anticipate and prevent potential vulnerable sites during the plasmid design phase.

Yet, over 100 IS*5* reads, detected with the help of the Genome ARTIST software, indicated that the pCYS_m plasmid displayed a high frequency of target site duplications with the sequence motif “ATAAAGCG”. The higher frequency could be explained by the fact that the pCYS_m plasmid, compared to the other two plasmids pCYS and pCYS_i, possesses an additional *cysM* gene for l-cysteine production, which imposes increased metabolic burden and thereby exerts enhanced evolutionary pressure on the cell population. Despite a thorough comparison between this motif and the IS*5* sequence, no similarities were detected, even at the crucial terminal inverted repeats responsible for target site recognition in other IS families. In contrast to IS*3*, the IS*5* family, which encompasses around 550 members, exhibits much higher diversity in terms of both sequence motifs and lengths [19]. Most of the members within this family are arranged in a single open reading frame that codes for the transposase. Interestingly, approximately 20% of the members in the IS5 family employ programmed transcriptional realignment frameshifting instead of the translational frameshifting mechanism observed in IS*3* members [16].

Future studies should explore whether certain DNA structure features outside the potential target site are involved in recognition. Furthermore, one could employ a plasmid reporter system that incorporates the “ATAAGCG” motif, enabling the activation of gene expression, such as GFP, upon IS5 transposition. Previous studies identified tetranucleotides with the motif “CTAG” as the consistent direct repeat or target site duplications for IS5, in contrast to the eight base pair repeats identified in this work [20,21].

**Figure 2:**
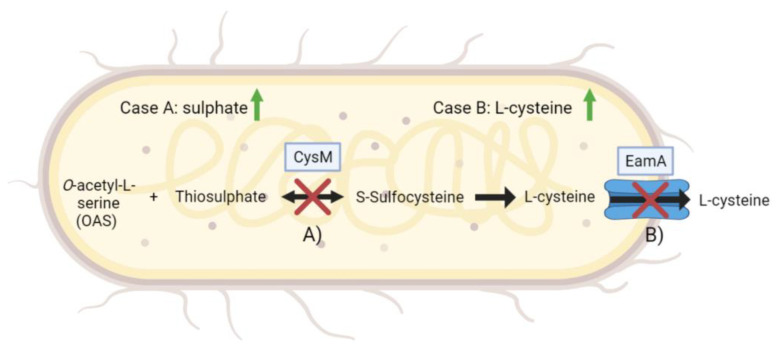
Potential effects on intracellular sulphate and l-cysteine level due to disruption of the synthetic l-cysteine pathway. Case A describes how intracellular sulphate level would increase when *cysM* is disrupted. O-acetylserine (OAS) and thiosulphate cannot be converted to S-sulphocysteine anymore. Case B highlights the effect of a disrupted l-cysteine exporter. EamA cannot transport l-cysteine anymore, which results in an increase in intracellular l-cysteine level. Both cases would be beneficial for the sulphur- and l-cysteine homeostasis of the cell.

The duplicated target sequence (TSD) traced the insertion of IS5 back to two sites within the pCYS_m plasmid: the open reading frame of the l-cysteine exporter EamA, and the ORF of Cysteine Synthase B (*cysM*) (Figure 2). If the exporter was impaired, the pCYS_m plasmid-harbouring populations would have accumulated l-cysteine within cells, enabling the cells to restore the sulphur balance by catabolizing larger quantities of l-cysteine. Alternatively, if the L-cysteine synthase was defective, the cells would have equally relieved the sulphur balance by directing the sulphur flow less towards l-cysteine and more towards other detrimental metabolic processes. Either scenario would suggest that *E. coli* populations with metabolic burdensome L-sulphur deprivation have undergone stress-induced adaptation via insertion sequences to increase cell viability and overall growth.

One of the extensively documented cases of IS activation resulting from environmental factors can be observed in the *glpFK/Crp* system of *E. coli*. This system exemplifies that the integration of an IS5 transposase into the *glpFK* promoter region can trigger the utilization of glycerol under conditions of starvation [22,23]. Similarly, in another instance, the typically dormant *bglGFB* operon, responsible for ß-glucoside utilization in *E. coli*, is stimulated by the insertion of an IS element upstream of the operon [24]. Humayun et al. propose a theoretical framework that elucidates how insertion events are facilitated at specific sites and how stress conditions are correlated with increased insertion at particular loci [20]. Notably, they demonstrate a connection between the occurrence of IS5 insertions in the *glpFK* and *bglGFB* cases and a specific DNA structure known as superhelical stress-induced duplex destabilization (SIDD). SIDD is employed as a bioinformatic model that evaluates the likelihood of denaturation of a given DNA sequence and generates an energy profile [25]. Regions with lower energy levels, indicating reduced stability, are considered less stable. Stress conditions negatively impact the linking number, a mathematical parameter that characterizes the degree of DNA twisting [26]. Consequently, regions with lower twisting properties, referred to as duplex destabilization sites, serve as potential hotspots for IS insertions. It is plausible that these destabilized DNA structures, located outside the target site duplications (TSDs), also influenced the observed insertion events in the present study.

As an alternative, future studies could consider utilizing a strain that no longer possesses highly active transposases. Umenhoffer et al. successfully engineered a minimal genome strain, MDS42, through a time-consuming process, which eliminated active transposases [27]. However, the deletion of additional genes, which were previously deemed non-essential, also altered the metabolic profile of the strain, leading to reduced chemical production efficiency. Therefore, a targeted deletion of selected active IS elements would be more advantageous. Moreover, an IS-inactivated *E. coli* strain, such as the DynaCompetent Cells IS-mutation Safe, could also be subjected to testing.

## Data Availability

All data and materials are available as described in the study and the Supplementary File.

## Supplementary Materials

The following supporting information can be downloaded at: https://www.mdpi.com/article/10.3390/genes14071317/s1, Figure S1: Per base coverage depths (x) of sequenced pCYS (A), pCYS_i (B) and pCYS_m (C) extracted from evolved populations (60 cell divisions) of *E. coli* W3110; Figure S2: Mapping of sequenced reads against insertion sequence (IS) families.

## References

[1] Aziz R.K., Breitbart M., Edwards R.A. (2010). Transposases are the most abundant, most ubiquitous genes in nature. Nucleic Acids Res..

[2] Hickman A.B., Dyda F. (2015). Mechanisms of DNA Transposition. Microbiol. Spectr..

[3] Lee H., Doak T.G., Popodi E., Foster P.L., Tang H. (2016). Insertion sequence-caused large-scale rearrangements in the genome of *Escherichia coli*. Nucleic Acids Res..

[4] Rugbjerg P., Myling-Petersen N., Porse A., Sarup-Lytzen K., Sommer M.O.A. (2018). Diverse genetic error modes constrain large-scale bio-based production. Nat. Commun..

[5] Heieck K., Arnold N.D., Bruck T.B. (2023). Metabolic stress constrains microbial L-cysteine production in *Escherichia coli* by accelerating transposition through mobile genetic elements. Microb. Cell. Fact..

[6] Drake J.W. (1998). Rates of Spontaneous Mutation. Genetics.

[7] Porse A., Gumpert H., Kubicek-Sutherland J.Z., Karami N., Adlerberth I., Wold A.E., Andersson D.I., Sommer M.O.A. (2017). Genome Dynamics of *Escherichia coli* during Antibiotic Treatment: Transfer, Loss, and Persistence of Genetic Elements In situ of the Infant Gut. Front. Cell. Infect. Microbiol..

[8] Gonçalves G.A.L. (2014). Evidence that the insertion events of IS2 transposition are biased towards abrupt compositional shifts in target DNA and modulated by a diverse set of culture parameters. Biotechnol. Prod. A Proc. Eng..

[9] Li H., Durbin R. (2009). Fast and accurate short read alignment with Burrows-Wheeler transform. Bioinformatics.

[10] Ecovoiu A.A., Ghionoiu I.C., Ciuca A.M., Ratiu A.C. (2016). Genome ARTIST: A robust, high-accuracy aligner tool for mapping transposon insertions and self-insertions. Mob. DNA.

[11] Chandler M., Fayet O., Rousseau P., Ton Hoang B., Duval-Valentin G. (2015). Copy-out-Paste-in Transposition of IS911: A Major Transposition Pathway. Microbiol. Spectr..

[12] Chandler M., Fayet O. (1993). Translational frameshifting in the control of transposition in bacteria. Mol. Microbiol..

[13] Rousseau P., Gueguen E., Duval-Valentin G., Chandler M. (2004). The helix-turn-helix motif of bacterial insertion sequence IS911 transposase is required for DNA binding. Nucleic Acids Res..

[14] Haren L., Normand C., Polard P., Alazard R., Chandler M. (2000). IS911 transposition is regulated by protein-protein interactions via a leucine zipper motif. J. Mol. Biol..

[15] Haren L. (1999). Integrating DNA: Transposases and Retroviral Integrases. Annu. Rev. Microbiol..

[16] Sharma V., Firth A.E., Antonov I., Fayet O., Atkins J.F., Borodovsky M., Baranov P.V. (2011). A pilot study of bacterial genes with disrupted ORFs reveals a surprising profusion of protein sequence recoding mediated by ribosomal frameshifting and transcriptional realignment. Mol. Biol. Evol..

[17] Sharma V., Prere M.F., Canal I., Firth A.E., Atkins J.F., Baranov P.V., Fayet O. (2014). Analysis of tetra- and hepta-nucleotides motifs promoting −1 ribosomal frameshifting in *Escherichia coli*. Nucleic Acids Res..

[18] Vögele K. (1991). High-level ribosomal frameshifting directs the synthesis of IS150 gene products. Nucleic Acid. Res..

[19] Mahillon J. (1998). Insertion Sequences. Microbiol. Mol. Biol. Rev..

[20] Humayun M.Z., Zhang Z., Butcher A.M., Moshayedi A., Saier M.H. (2017). Hopping into a hot seat: Role of DNA structural features on IS5-mediated gene activation and inactivation under stress. PLoS ONE.

[21] Siguier P., Gourbeyre E., Chandler M. (2014). Bacterial insertion sequences: Their genomic impact and diversity. FEMS Microbiol. Rev..

[22] Zhang Z., Saier M.H. (2009). A mechanism of transposon-mediated directed mutation. Mol. Microbiol..

[23] Zhang Z., Saier M.H. (2009). A novel mechanism of transposon-mediated gene activation. PLoS Genet..

[24] Schnetz K. (1992). IS5: A mobile enhancer of transcription in *Escherichia coli*. Proc. Natl. Acad. Sci. USA.

[25] Fye R.M. (1999). Exact method for numerically analyzing a model of local denaturation in superhelically stressed DNA. Phys. Rev. E.

[26] Sheridan S.D., Benham C.J., Hatfield G.W. (1998). Activation of gene expression by a novel DNA structural transmission mechanism that requires supercoiling-induced DNA duplex destabilization in an upstream activating sequence. J. Biol. Chem..

[27] Umenhoffer K., Feher T., Baliko G., Ayaydin F., Posfai J., Blattner F.R., Posfai G. (2010). Reduced evolvability of *Escherichia coli* MDS42, an IS-less cellular chassis for molecular and synthetic biology applications. Microb. Cell. Fact..

