# Supplementary material for: Localization of Insertion Sequences in Plasmids for L-Cysteine Production in E. coli
Source: Genes (Basel). 2023 Jun 22;14(7):1317. doi: 10.3390/genes14071317 (PMC10379815; doi:10.3390/genes14071317)
Supplement: Supplementary file 1 [file genes-14-01317-s001.zip › genes-2464214-supplementary.pdf]

Localization of Insertion Sequences in Plasmids for L-Cysteine Production in *E. coli*

Kevin Heieck and Thomas Brück *

Chair of Synthetic Biotechnology, School of Natural Sciences, Technical University of Munich, Lichtenbergstraße 4, 85748 Garching, Germany; kevin.heieck@tum.de
* Correspondence: brueck@tum.de; Tel.: +49-89-289-13253

A

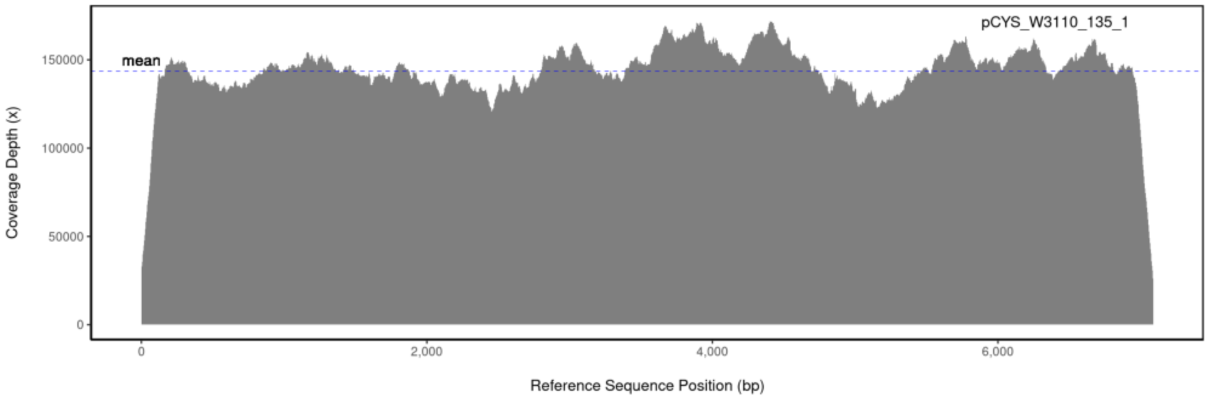

B

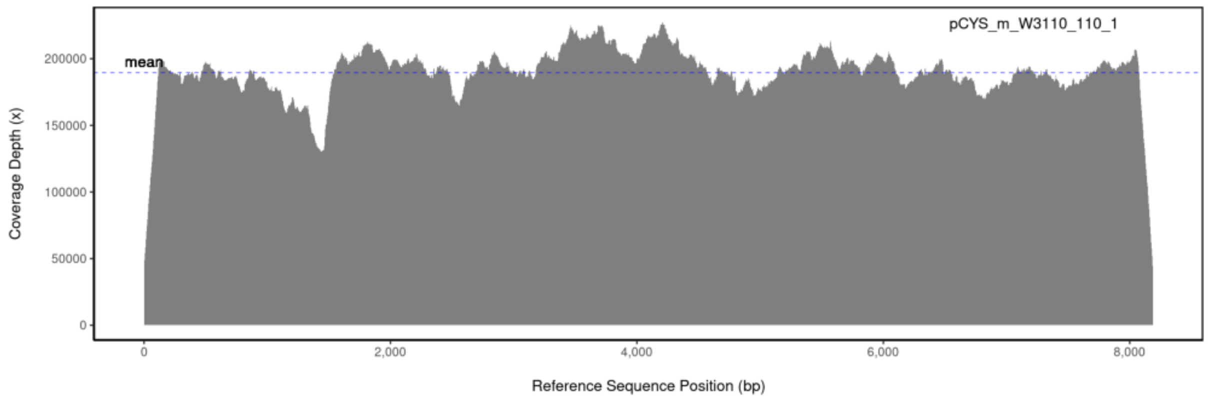

C

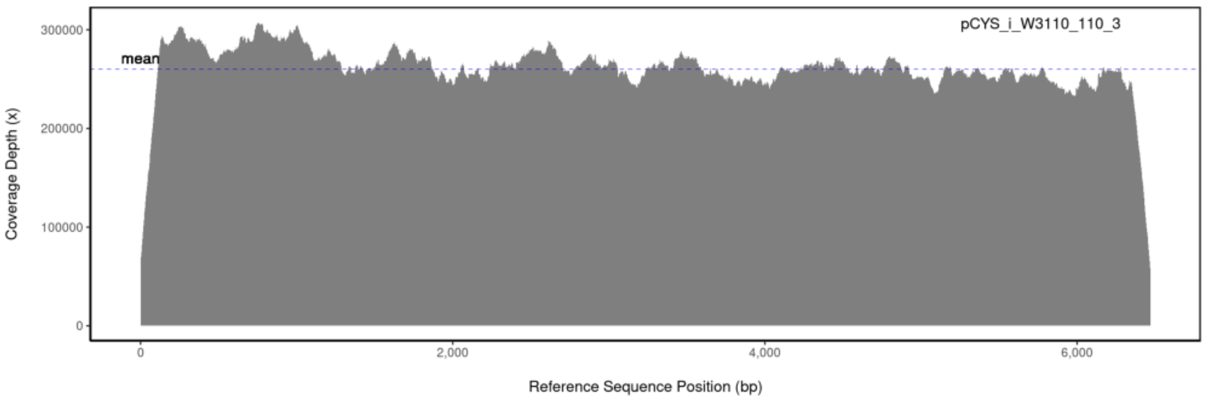

Supplementary Figure S1: Per base coverage depths (x) of sequenced pCYS (A), pCYS_i (B) and pCYS_m (C) extracted from evolved populations (60 cell divisions) of E. coli W3110. Sequencing was conducted with Illumina Novaseq paired end 2x150bp.

Plasmid	pCYS				pCYS_i				pCYS_m			
**Strain**	**W3110**		**MDS42**		**W3110**		**MDS42**		**W3110**		**MDS42**	
**Population**	**EGP**	**LGP**	**EGP**	**LGP**	**EGP**	**LGP**	**EGP**	**LGP**	**EGP**	**LGP**	**EGP**	**LGP**
Unmapped reads to plasmid sequence [%]	4.4	8.2	2.7	2.5	3.3	6	2.5	2.4	3.8	7.1	2	2.1
Reads mapped to IS [%]	0.13	0.24	0.01	0.01	0.11	0.18	0.01	0.01	0.12	0.23	0.01	0.01
Reads mapped to IS [total]	3803	5852	271	249	2361	5253	261	245	2101	6070	180	196

Supplementary Figure S2: Mapping of sequenced reads against insertion sequence (IS) families [1].: Table showing the percentages of reads that did not map to the plasmid sequences as well as the percentages of those unmapped reads mapped to IS. Plasmids pCYS, pCYS_i and pCYS_m extracted from evolved populations (60 cell divisions) of W3110 got deep sequenced (Methods).

Reference

1. Heieck, K.; Arnold, N.D.; Bruck, T.B. Metabolic stress constrains microbial L-cysteine production in *Escherichia coli* by accelerating transposition through mobile genetic elements. *Microb. Cell. Fact.* **2023**, *22*, 10. https://doi.org/10.1186/s12934-023-02021-5.
